# The limits of curiosity? New evidence for the roles of metacognitive abilities and curiosity in learning

**DOI:** 10.1007/s11409-024-09407-9

**Published:** 2024-11-15

**Authors:** Xiaoyun Chen, Katherine E. Twomey, Miranda Hayes, Gert Westermann

**Affiliations:** 1 Department of Psychology, D6, Fylde College, Lancaster University, Lancaster, LA1 4YF UK; 2 Division of Human Communication, Development and Hearing, University of Manchester, Manchester, UK

**Keywords:** Curiosity, Confidence, Prior knowledge, Metacognition

## Abstract

The current study investigated how metacognitive abilities such as Knowledge Confidence (subjective prior knowledge estimate) and Correctness Confidence (appraisal of the likelihood of closing the gap) relate to curiosity, and examined the roles of these metacognitive measures and curiosity in learning. Using a blurred picture paradigm in which participants viewed blurred pictures and provided their metacognitive and curiosity estimates, the current study identified distinct connections between Knowledge Confidence, Correctness Confidence and curiosity. Our finding suggests that when Knowledge Confidence is at low or medium levels, curiosity is particularly heightened. In contrast, Correctness Confidence linearly influences curiosity such that the higher the Correctness Confidence, the greater the curiosity. We also find that learning is best predicted by a learner’s metacognitive appraisal of their knowledge gap, especially when they are on the verge of knowing, and this learning effect is independent of curiosity. These findings provide new evidence for the role of curiosity and metacognition in learning, highlighting limits of the effect of curiosity on learning.

**Supplementary Information:**

The online version contains supplementary material available at 10.1007/s11409-024-09407-9.

## Introduction

### Curiosity and metacognitive abilities

Curiosity is the intrinsic desire to acquire information and to explore the environment for understanding of the world (Kidd & Hayden, 2015; Vogl et al., 2020). Curiosity-based learning involves not only novel information, but also extends to information about which we are uncertain, with the resolution of this uncertainty updating our current knowledge representations or understanding (Berlyne, 1954, 1972). As a key driver of knowledge acquisition, curiosity has been empirically demonstrated to boost learning and enhance the memory of both task materials and unrelated items (Berlyne & Normore, 1972; Chen et al., 2022; Fandakova & Gruber, 2021; Gruber et al., 2014; Jepma et al., 2012; Kang et al., 2009; Wade & Kidd, 2019). This beneficial effect of curiosity on learning also persists over time (Fastrich et al., 2018; Stare et al., 2018). Given the central role of curiosity in boosting learning outcomes, identifying facilitators of curiosity may have great educational implications and consequently, the field of curiosity research is growing.

Metacognition, especially metacognitive appraisal in evaluating one’s subjective prior knowledge state, is one of the factors that trigger curiosity, and it substantially influences subsequent information-seeking behaviour (Litman, 2009; Loewenstein, 1994; Metcalfe et al., 2020). This idea was first explicitly introduced in the *information gap* theory (Loewenstein, 1994) in which curiosity is defined as a cognitive desire that arises from the perception of a gap in knowledge and understanding. Curiosity stems from an individual’s awareness of the gap between what one knows and what one wants to know. In other words, from this perspective at least two prerequisites are emphasized in triggering curiosity: the learner’s subjective estimation of their current level of knowledge, and the identification of a piece of specific information for closing the knowledge gap.

**Fig. 1:**
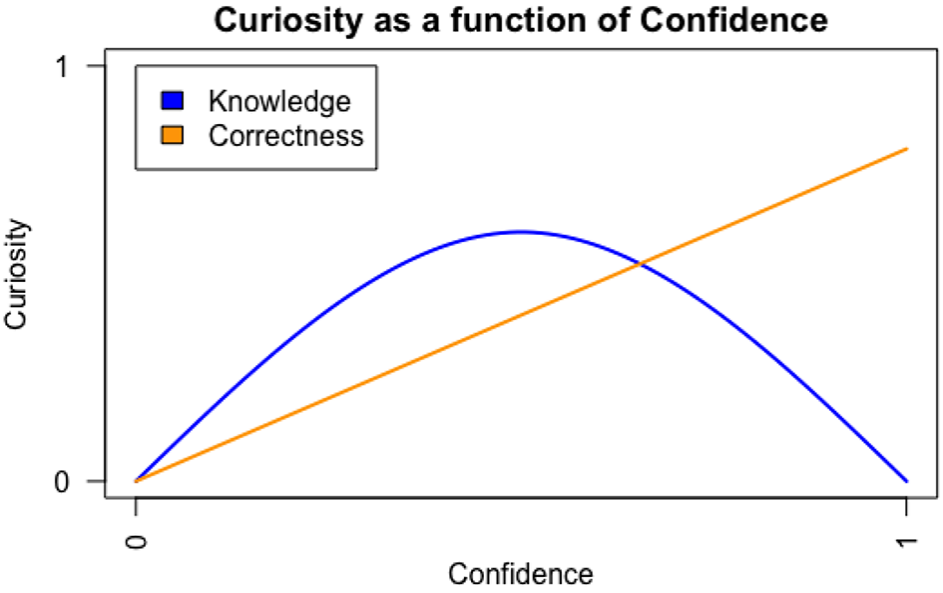
A simulated figure: curiosity as a function of knowledge confidence and correctness confidence

On this information gap account, the learner’s subjective appraisal of prior knowledge reflects confidence in their existing knowledge and could be an index of the learner’s strength of knowledge on the topic. Hence, we refer to this subjective appraisal of prior knowledge as *Knowledge Confidence*. Knowledge Confidence is theoretically assumed to have an inverted U-shaped relationship with curiosity (Fig. 1). When a learner believes they possess the knowledge to solve the task at hand (i.e., the ‘*I Know*’ state; Litman, 2009), little curiosity associated with information-seeking behaviour is induced as there is no new knowledge needed. When a learner thinks they do not possess the knowledge at all, this corresponds to the ‘*I Don’t Know*’ state in which less curiosity and fewer information-seeking behaviours are provoked, as the knowledge gap is too large and the desired knowledge will not be accessible. More specifically, the ‘*I Don’t Know*’ state has less uncertainty as the learner is certain that they do not know (Brooks et al., 2021; Loewenstein, 1994; Metcalfe et al., 2020). However, when a learner appraises that they have some knowledge but are uncertain whether their current knowledge is correct, it creates a ‘*Not Sure*’state or related ‘*Feeling-of-Knowing*’ state, which elicits more curiosity and exploratory behaviours in order to obtain the desired information (Brooks et al., 2021; Hanczakowski et al., 2014; Litman et al., 2005; Metcalfe et al., 2017). In line with this theoretical assumption, Kang and colleagues (2009), using a trivia question paradigm, found that participants’ curiosity peaked when they had an intermediate level of Knowledge Confidence (equivalent to participants’ maximal uncertainty as to whether they knew the answer). Similar findings were reported also in Baranes et al. (2015), Dubey and Griffiths (2020) and Metcalfe et al. (2020).

**Fig. 2:**
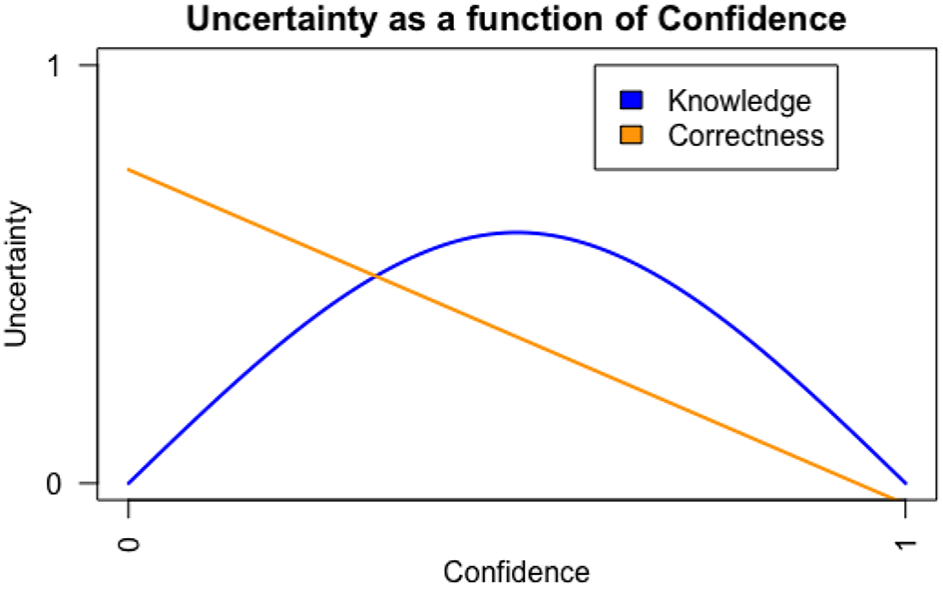
A simulated figure: uncertainty as a function of knowledge confidence and correctness confidence

Of relevance, as compared to Knowledge Confidence, which is traditionally linked to curiosity, other evidence suggests that there is also another type of confidence appraisal that influences curiosity in a different way– *Correctness Confidence*. Knowledge Confidence is different from Correctness Confidence. The former emphasises the evaluation of one’s general knowledge state (i.e., Do I know this topic or not?), whereas the latter is the appraisal of the likelihood to close the gap successfully (i.e., whether my guess is correct or not). In other words, Knowledge Confidence reflects a general sense of knowledge, whereas Correctness confidence reflects appraisal of a specific prediction. Correctness Confidence is thought to vary in a (negative) linear fashion with uncertainty about the information/answer. For example, if a learner has zero confidence in being correct, the uncertainty about the answer is high; if a learner has zero confidence in their knowledge, the uncertainty is low as the learner should be certain that they do not have the answer (Fig. 2).

Most importantly, it is not yet clear how Correctness Confidence is related to curiosity. Previous studies that measured confidence without specifying its type, found that it predicts curiosity linearly (Fig. 2) such that higher Correctness Confidence is associated with greater curiosity (i.e., confirmation curiosity), due to the desire to confirm or verify predictions. For example, Wade and Kidd (2019), using a trivia question paradigm, found that participants were more curious about the questions when they believed their predictions were correct (high Correctness Confidence). Theobald et al. (2022), using pupil dilation as an index of curiosity, found that participants’ pupil size increased when seeing trivia questions about which they were more confident in knowing the correct answer relative to questions attracting less confidence. Taken together, these studies suggest that different types of confidence may mediate curiosity in a variety of ways.

### Confidence, curiosity and learning

Confidence as a part of metacognitive appraisal not only reflects subjective epistemic states but also influences learning. It has been found that high Correctness Confidence benefits learning as compared to low Correctness Confidence (Metcalfe & Miele, 2014). This enhancing effect of Correctness Confidence on learning may be due to incorrect predictions made with high confidence eliciting surprise reactions, which increases attention to the correcting information and results in enhanced memory for that information (Metcalfe, 2017). On the other hand, high Correctness Confidence might reflect a high degree of familiarity with the information. Updating high-confidence error responses when the correct information is already stored (but was not retrieved correctly) may be relatively easy compared to low-confidence error responses (Butterfield & Metcalfe, 2006). Although it is evident that confidence can influence curiosity and learning, there has been a lack of investigations comparing the various roles of confidence in curiosity as well as the effects of curiosity and confidence on learning.

The current study set out to examine the two distinct types of confidence we proposed - Knowledge Confidence (i.e., subjective prior knowledge estimate) and Correctness Confidence (i.e., appraisal of the likelihood of closing the gap), and their associations with curiosity. We then investigate the roles of two types of confidence and curiosity in learning. Importantly, because the current curiosity and metacognition literature predominantly uses linguistically-mediated information such as trivia questions, more research in diverse contexts and with different paradigms is necessary to complement existing findings. Therefore, in the attempt to extend the generalisability of research findings in the field by using different types of stimuli, we used blurred images of day-to-day objects and living creatures across a wide range of categories to induce curiosity. Blurred images have been reliably found to elicit curiosity in the literature (Nicki, 1970; Jepma et al., 2012).

In this study, participants were presented with a series of blurred images to evoke their curiosity. For each blurred image, participants were asked if they knew what it was (Knowledge Confidence), to provide a best guess, then rated their confidence in their guess (Correctness Confidence) as well as their level of curiosity about the image. We hypothesized that Knowledge Confidence would show an inverted U-shaped relationship with curiosity, and Correctness Confidence would show a linear relationship with curiosity. Second, to examine the roles of these metacognitive abilities and curiosity in learning, participants completed a surprise memory recall test. After answering the questions about each blurred image, they were shown all blurred images again and were asked to recall as many correct answers as they could. Recall accuracy was viewed as a learning outcome. As both curiosity and confidence have substantial impacts on learning, we hypothesised that curiosity and confidence would be associated with better memory recall performance.

## Methods

### Participants

A total of 108 participants, recruited online from Sona Systems (https://www.sona-systems.com) and Prolific (https://prolific.ac), took part in the online experiment on the Gorilla online experimental platform (www.gorilla.sc). The sample size was calculated using G*Power based on Wade and Kidd (2019) with an effect size of *r* =.40 in a correlational model with 0.95 power and 0.05 alpha. The power analysis suggested a sample size of 71 participants. We tested 108 participants to sufficiently detect the effect. Eight participants were excluded for providing the same curiosity rating on at least 90% of trials in the learning phase, resulting in 100 participants (*M*_*age*_ = 22.64, *SD*_*age*_ = 7.60, *N*_*female*_ = 74) in the final analysis. Participants received either university course credits or monetary rewards (£10 per hour). Participants were given information about the study and provided informed consent before participation. The browser for the online task was limited to Google Chrome only as it has been shown that Google Chrome is more compatible with running an experiment in Gorilla (Anwyl-Irvine et al., 2021) compared to other types of browsers. The device was limited to laptops only for the same reason. The study received ethics approval from Lancaster University in the UK.

### Materials

All stimuli were adapted from Moreno-Martínez and Montoro’s (2012) database of 360 high-quality colour images. Stimuli consisted of 60 clear, 450 by 350-pixel object images of animals, food, instruments, furniture, utensils and vehicles, placed centrally on a grey background. As previous curiosity literature highlights the role of an intermediate level of uncertainty in inducing curiosity (Berlyne & Normore, 1972; Jepma et al., 2012; Nicki, 1970), following this literature we blurred these stimuli with a 30-degree (medium) Gaussian filter in Matlab (Version R2016b), resulting in 60 blurred and 60 corresponding clear images (see Table 1 for examples).

**Table 1:**
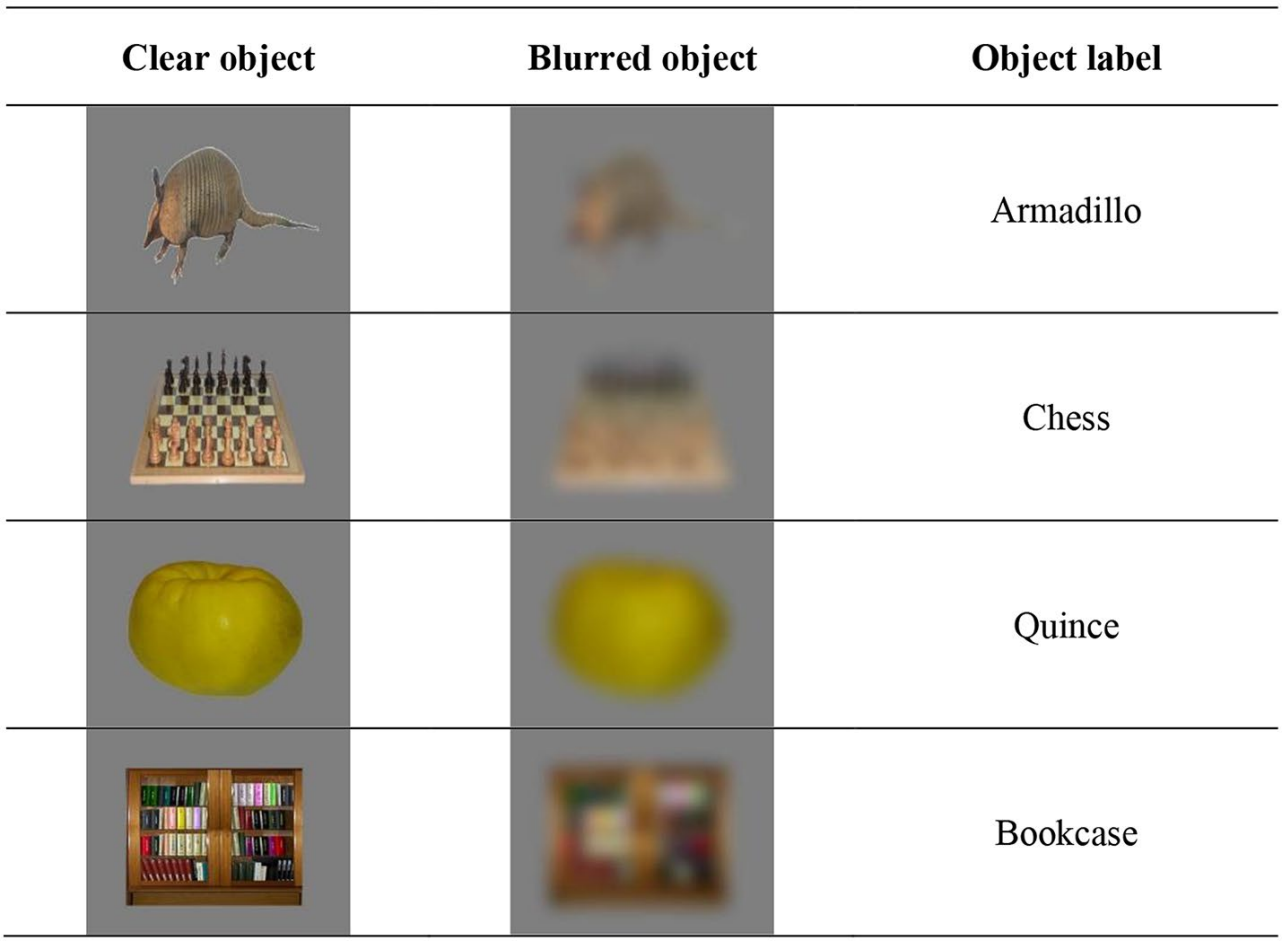
Exemplars of the object images with their blurred versions and corresponding labels

### Task design

**Fig. 3:**
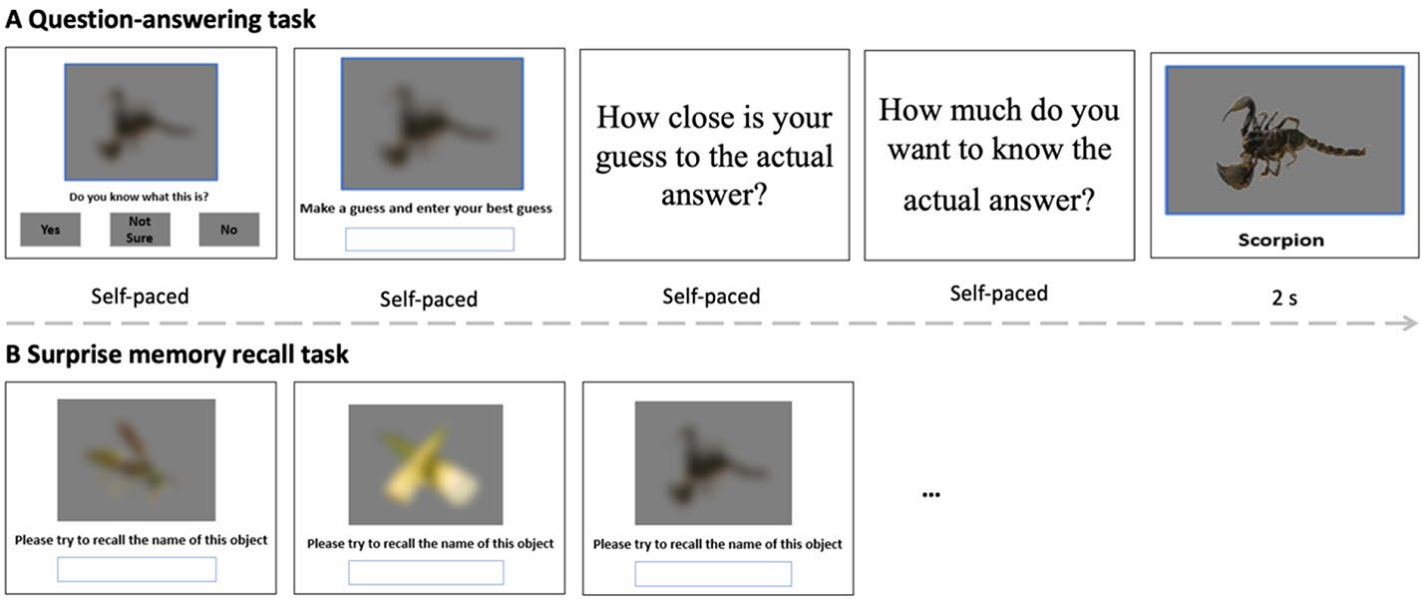
**A**: Trial structure of the question-answering task (60 trials). **B**: The surprise memory recall task after the question-answering task

This experiment consisted of two tasks (see Fig. 3): a question-answering task and a surprise memory recall task. In the question-answering task, participants were presented with 60 blurred images, one image at a time. For each image, participants were asked the following questions in sequence: (1) Knowledge Confidence. Participants saw the question “*Do you know what this is?*”, and were asked to give their response by clicking one of three response buttons: “*Yes”* if they were sure they knew the answer, *“No”* if they did not know the answer, or “*Not Sure*” if they were not sure about the answer. (2) providing a guess: participants were asked to make a best guess and type their guess into a box; (3) Correctness Confidence: participants were asked the question “*How close is your guess to the actual answer*?” and to rate their confidence in their guess on a scale from 1 (Not at all) to 6 (Very close); (4) curiosity: participants rated their curiosity by answering the question “*How much do you want to know the actual answer?*” on a scale from 1 (Not at all) to 6 (Very much). All the questions were self-paced. As soon as participants responded, the task automatically proceeded. In each trial, after participants responded to the curiosity question, a clear image corresponding to the blurred image with its label was presented for 2 s.

Immediately after the question-answering task, participants were asked to complete a surprise memory recall task in which all the blurred images from the question-answering task were presented again, one image at a time. Participants were asked to recall the name of each image by typing their answers into a box. The order of the stimuli was randomised across participants and phases.

### Analysis

Raw data were exported from Gorilla and imported to RStudio (Version 1.3.1093) for cleaning and analysis. Each participant provided guesses and ratings for 60 trials, resulting in a total of 6000 trials. All 6000 trials were included to examine the relationships between Knowledge Confidence, Correctness Confidence and curiosity. For predicting recall accuracy, trials were excluded if the guesses in the question-answering task were correct (*N* = 1980 trials), or not appropriate (e.g., “*?*”, “*no idea*”; *N* = 8 trials), resulting in 4010 trials for statistical analysis (66.83% of all 6000 trials). We excluded the correct trials because we were interested in participants’ learning of objects for which they made an initial wrong guess.

The accuracy of the guesses from the question-answering task as well as the responses from the surprise memory recall task were judged by three raters independently. The three raters were asked to judge if a participant’s response was the same as the correct label. If a response was too generic (‘*animal’* for rabbit), too vague (‘*a fruit that I did not know existed*’ for lemon) or lacked content (‘*??*’ or “*no idea*”), it was scored as incorrect. If a response included an obvious typing mistake (‘*rebbit*’ for rabbit) or had different labels with the same meaning (‘*bookshelf*’ for bookcase), it was marked as a correct response. Responses were accepted as correct only if they were rated as correct by at least two raters. The reliability of agreement for multiple raters was assessed using Fleiss’ kappa analysis (Falotico & Quatto, 2015; Fleiss et al., 2013) using the ‘irr’ package (Gamer et al., 2019) in R. A Fleiss’ kappa value greater than 0.75 is taken to represent high agreement. There was excellent agreement (*kappa* = 0.83, *p* <.0001), suggesting high inter-rater reliability between the three raters.

Statistical models were fitted accordingly to answer the questions. For ease of interpretation, the respective analysis and the associated results will be presented together below. The associated R code can be found on OSF: [link]

## Results

### Relationships between curiosity and confidence

To evaluate the extent to which participants’ curiosity ratings varied with their Knowledge Confidence and Correctness Confidence, we fitted a linear mixed-effects model using lme4 (Bates et al., 2015) in R. The initial model included Knowledge Confidence as a nominal variable, Correctness Confidence as a continuous variable and their interactions as fixed effects, and participant and stimulus as random effects. However, the interaction term was not a significant predictor (*p* =.33), which was determined by dropping each predictor from the full model one at a time. Therefore, the interaction term was not included in the final full model. To avoid multiple testing (Schielzeth & Forstmeier, 2009), the full model was compared with a null model consisting of only the random effect terms from the full model using a likelihood ratio test. In addition, collinearity was examined using Variance Inflation Factors (VIF) from the car package (Fox et al., 2022), suggesting that there were no collinearity issues (Table S1 in Supplementary Materials).

Full model 1 structure Curiosity ~ Knowledge Confidence + Correctness Confidence + (1| Participant) + (1| Stimulus).

Null model 1 structure Curiosity ~ 1 + (1| Participant) + (1| Stimulus).

**Fig. 4:**
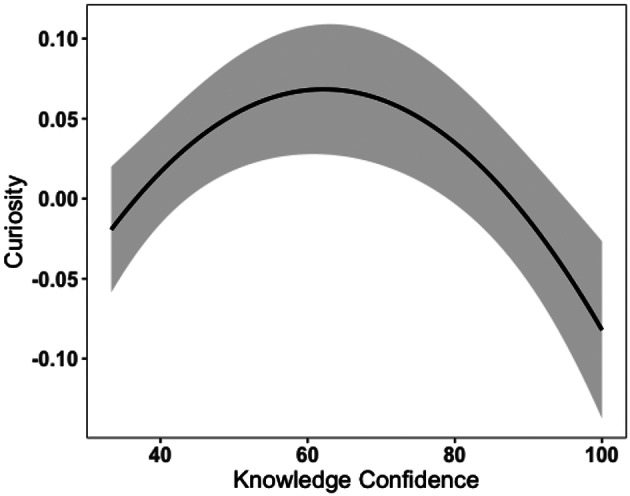
The relationship between Knowledge confidence and curiosity. *Note*: For the purpose of data visualisation as well as visual comparison with Fig. 5 and previous research (Kang et al., 2009), Curiosity was normalised and Knowledge Confidence was converted to a100 point scale. The scale of converted Knowledge Confidence ranges from 33.33 to 100. Our data suggested that low and intermediate levels of Knowledge Confidence were positively associated with curiosity whereas high Knowledge Confidence were negatively associated with curiosity

Results of the full-null model comparison indicate that Full Model 1 provided a good fit (*χ*^*2*^ = 20.30, *df* = 3, *p* <.001, *R*^*2*^ *=* 0.40), and revealed significant fixed effects on curiosity rating (Table 2). Specifically, Knowledge Confidence was significantly associated with curiosity. Both Not Sure (*β* = 0.12, *SE* = 0.04, *p* =.001) and No (*β* = 0.11, *SE* = 0.05, *p* =.041) responses positively predicted cutiosity as compared to Yes response (Fig. 4). This result indicated that the Not Sure and No responses for Knowledge Confidence had a similar effect on curiosity. This outcome is inconsistent with the information-gap theory (Loewenstein, 1994) which predicts that curiosity should peak when Knowledge Confidence is at a moderate level (Knowledge Confidence = 0.5), reflecting a maximal level of uncertainty (Kang et al., 2009). In other words, either low (No) or high (Yes) Knowledge Confidence would suggest low uncertainty and thus should be associated with low curiosity. However, our data suggested that participants were more curious when they did not know at all about the blurred picture and when they were unsure about the answer before making an explicit guess.

**Fig. 5:**
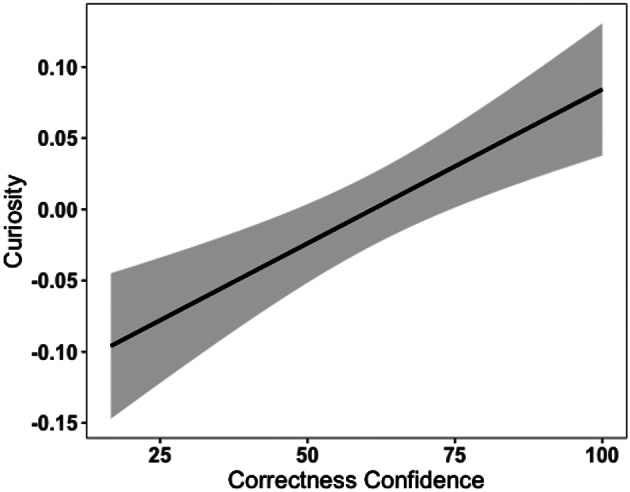
The relationship between correctness confidence and curiosity. *Note*: For the purpose of data visualisation as well as visual comparison with Fig. 4, Curiosity was normalised and Correctness Confidence was converted to a 100 point scale. Our data suggested that Correctness Confidence had a linear relationship with curiosity

Similarly, Correctness Confidence also had a significant effect on curiosity (*β* = 0.05, *SE* = 0.01, *p* <.001), suggesting a linear relationship. Figure 5 depicts this relationship. In line with our prediction, this result suggests that Correctness Confidence linearly predicts curiosity such that higher Correctness Confidence is associated with higher curiosity. In other words, participants were most curious about the blurred picture (i.e., the identity/actual answer) when they were more confident with their guess being correct.

**Table 2:** Linear mixed-effects model estimates of full model 1

Term	$\:\beta\:$	SE	t	*p*	95% CI
(Intercept)	4.19	0.11	38.94	< 0.001***	[0.72, 0.96]
Knowledge Confidence: Not Sure	0.12	0.04	3.23	0.001**	[0.03, 0.12]
Knowledge Confidence: No	0.11	0.05	2.05	0.041*	[1.01, 1.04]
Correctness Confidence	0.05	0.01	3.97	< 0.001***	[3.98, 4.40]

*Note* *<0.05, **p <.01, ***p <.001

### What predicts learning?

To investigate whether curiosity, Knowledge Confidence and Correctness Confidence influenced participants’ learning of names of the blurred objects, only the images with incorrect guesses in the question-answering task (i.e., the rating phase) were included in this analysis. Raw accuracy in the question-answering task before excluding all correct trials was 33.17%, suggesting the difficulty of the task was at a reasonable level.

A binomial Generalised Logistic Mixed-Effects Model (GLMM) was fitted (Baayen, 2008) using the lme4 package (Bates et al., 2015). The full model included curiosity, Knowledge Confidence, Correctness Confidence and their interactions as fixed effects and the individual participant and stimulus as random effects. The significance of predictors was determined by dropping each predictor from the full model one at a time. The interaction term was not a significant factor (*p =*.52) and was therefore not included in the final full model to ease computation. Results and a summary of the comparison model with the interaction term included is presented in Supplementary Information in Table S3. As distribution of curiosity and Correctness Confidence were approximately normal (Robitzsch, 2020; Snijders & Bosker, 2011), curiosity and Correctness Confidence were fitted to the final model as continuous variables after being *z*-transformed to ease model convergence and make model interpretation easier. One theoretically identifiable random slope component (Correctness Confidence term within-participant) was included to avoid an overconfident model and inflation of the type I error rate (Barr et al., 2013; Schielzeth & Forstmeier, 2009). The full model was compared with a null model consisting of only the same random effect terms as the full model. Additionally, there were no serious collinearity issues found (Table S2 in Supplementary Materials). Confidence intervals (95%) were derived using the function bootMer from the lme4 package with 1000 parametric bootstraps.

Full model 2 structure Recall Accuracy ~ Curiosity + Knowledge Confidence + Correctness Confidence + (1 + Correctness Confidence| Participant) + (1| Stimulus), family = binomial.

Null model 3 structure: Recall Accuracy ~ 1 + (1 + Correctness Confidence| Participant) + (1| Stimulus), family = binomial.

Results of the full-null model comparison revealed a significant improvement in the full model (*χ*^*2*^ = 12.00, *df* = 4, *p* =.017, *R*^*2*^ *=* 0.39) and a significant effect of Knowledge Confidence on recall accuracy (Table 3). More specifically, the *Not Sure* response of Knowledge Confidence was positively associated with recall accuracy (*β(logit)* = 0.29, *SE* = 0.11, *z* = 2.71, *p* =.007). In contrast to previous literature, curiosity did not have a significant effect on recall accuracy (*β(logit)* = 0.07, *SE* = 0.05, *z* = 1.38, *p* =.17). Different from our hypotheses, Correctness Confidence did not have a significant impact on test recall accuracy (*β(logit)* = 0.03, *SE* = 0.06, *z* = 0.58, *p* =.56).

**Table 3:** Estimates from the binomial generalised linear mixed model (full model 2) predicting test recall accuracy

Terms	$\:\beta\:$	SE	95% CI	z	*p*
Intercept	0.56	0.20	[0.17, 0.97]	-	-
Correctness Confidence	0.03	0.06	[-0.08, 0.14]	0.58	0.56
Curiosity	0.07	0.05	[-0.03, 0.16]	1.38	0.17
Knowledge Confidence: NotSure	0.29	0.11	[0.08, 0.51]	2.71	0.007*
Knowledge Confidence: Yes	0.15	0.15	[-0.15, 0.44]	0.99	0.32

*Note* *p <.05; **p <.01

## Discussion

The current study indicates that curiosity is influenced by metacognitive appraisal: Knowledge Confidence and Correctness Confidence. However, the nature of their individual connections with curiosity varies in distinct ways. Interestingly, our results also show that learning is independent of curiosity and is best predicted by a learner’s Knowledge Confidence, especially when the learner is most uncertain about their knowledge. Overall, these findings help disentangle the roles of metacognitive abilities in curiosity and help clarify the effects of curiosity and different types of confidence on learning, providing new evidence to this field.

We identified two separable types of metacognitive confidence and investigated their relationships with curiosity: Knowledge Confidence and Correctness Confidence. We found that the relationship between Knowledge Confidence and curiosity may not be characterised by a quadratic pattern (i.e., inverted U-shaped) as previous literature suggested. Our results revealed that when Knowledge Confidence is at low and medium levels, curiosity is particularly heightened. In contrast, Correctness Confidence influences curiosity in a linear way such that the higher the Correctness Confidence, the greater the curiosity.

Knowledge Confidence reflects subjective uncertainty in evaluating one’s strength of knowledge on the topic. When learners are either highly confident or have no confidence that they possess the knowledge, subjective uncertainty about their knowledge state remains low, resulting in low curiosity. When learners are unsure and appraise that the information is slightly outside their current knowledge, it creates an uncertain state “*Not Sure*” similar to on-the-verge-of-knowing (Litman et al., 2005; Litman, 2009) that represents maximal uncertainty in their current knowledge (Nicki, 1970; Berlyne, 1972). It is thought that such a state involves partial retrieval of information from memory. The evaluation of and selection between the retrieved alternatives may result in greater uncertainty, which requires additional cognitive processes and greater motivation to resolve the cognitive conflicts, leading to higher curiosity to optimize their learning (Metcalfe et al., 2020; Oudeyer et al., 2016).

Interestingly, unlike the ‘*Not Sure*’ state with maximal uncertainty, low Knowledge Confidence (i.e., ‘*I Don’t Know*’ state) is thought to be associated with less curiosity due to unsuccessful information retrieval yielding a knowledge gap that is too large to eliminate (Loewenstein, 1994). However, our findings also suggest that low Knowledge Confidence is linked to higher curiosity ratings as compared to high Knowledge Confidence (Fig. 4). This might be due to the nature of stimuli used in the current study, specifically, images of objects. As humans are exposed to an enormous amount of visual input, participants’ default belief with regards to an object might be ‘*I must have seen this object’* (as a relatively strong prior), but the possibility of the identities of the object could be numerous, leading to larger outcome uncertainty that increases curiosity (van Lieshout et al., 2018). It is also possible that low Knowledge Confidence is associated with a large knowledge gap and as compared to no knowledge gap, it still drives curiosity to obtain new information (Berlyne et al., 1963; Dubey & Griffiths, 2020).

Different from Knowledge Confidence, Correctness Confidence evaluates one’s belief in the correctness of one’s knowledge which is associated with desires either to confirm (i.e., ‘*was I correct?*’) or to verify (i.e., whether I was correct or not) predictions as a way of updating one’s prior schema of the world. In other words, the more Correctness Confidence in a prediction, the more curiosity would be provoked. This result is consistent with the findings from Wade and Kidd’s study (2019), suggesting that a desire to verify or confirm one’s predictions triggers higher curiosity (e.g., ‘*I am confident that my guess is correct*,*therefore*,*I am more curious about the answer’*). It is possible that by having a prediction in mind, it increases the state of curiosity (i.e., greater attentional arousal) indicated by larger pupil dilation as compared to not having a prediction (Brod & Breitwieser, 2019). It has also been suggested that making a prediction generates or increases a relevant knowledge gap, which in turn increases curiosity, motivating verification and confirmation of the prediction (Loewenstein, 1994).

Another possible reason for this linear relationship could lie in the paradigm itself. When seeing and being asked about the identity of a blurred object, the answer could be associated with many unspecific objects that look alike. The search spaces for the identity would be enlarged, making it difficult to associate with related semantic memory. In other words, even though participants have high Correctness Confidence, the related Knowledge Confidence is not high due to larger outcome uncertainty (i.e., myriad identities for a given blurred object) leading to greater curiosity. Moreover, compared to trivia questions, when using blurred object images to induce curiosity, participants might have a stronger default belief to the blurred objects due to their everyday visual experiences with objects. Having a stronger prior might bias decision-making towards confirmation of predictions, whereas larger outcome uncertainty motivates curiosity to reduce the uncertainty. Further studies could manipulate the degree of familiarity of blurred objects in relation to confirmation bias to verify this possibility.

We also examined whether curiosity, Knowledge Confidence, and Correctness Confidence affected memory recall accuracy of the task materials. Surprisingly, in contrast to previous literature (Baranes et al., 2015; Brod & Breitwieser, 2019; Gruber et al., 2014; Jepma et al., 2012; Kang et al., 2009; Wade & Kidd, 2019), curiosity did not obviously affect recall accuracy. Instead, recall accuracy was best predicted by Knowledge Confidence, such that the ‘*Not Sure*’ state of Knowledge Confidence was related to higher recall accuracy. Unlike previous studies where the combined effect of knowledge states and curiosity on learning was highlighted (Brooks et al., 2021; Hanczakowski et al., 2014; Litman et al., 2005), our data suggest that there is no interaction effect on learning between Knowledge Confidence and curiosity. Instead, our finding suggests Knowledge Confidence (medium level) alone predicted better learning.

This result could be explained by the region of proximal learning framework (Kornell & Metcalfe, 2006; Metcalfe, 2009). According to this framework, the judgement of metacognitive states could lead to effective learning such that learners would focus on learning the easiest information they do not know over the already known or the most difficult information. In our case, the metacognitive state of ‘*Not Sure*’ indicates that a learner is in the ‘optimal learning zone’ where they can focus on and prioritize learning information that is on the verge of being known, resulting in effective learning (Metcalfe et al., 2020). Although the region of proximal learning framework is often applied to intentional learning, the question-asking task in this study was likely to provoke similar processes underlying the proximal learning framework in a passive way. In the current design, participants were asked to think about their current knowledge and make a prediction as well as to evaluate their confidence in correctness. These questions might create a spill-over effect that makes participants unintentionally maximise their learning efficiency by focusing on materials that offer the greatest potential for learning gains. In addition, there might also be other potential frameworks that may explain the results not from curiosity but other motivational factors such as self-efficacy (i.e., the belief in one’s ability to perform or compete a task), providing a direction for future investigations.

Overall, extending the current literature, this is the first study to identify two different types of metacognitive abilities, i.e., Knowledge Confidence and Correctness Confidence, and their distinct relationships with curiosity. These findings reveal the diversity of objectives of learners’ curiosity, including motives for resolving the knowledge gap as well as confirmation of predictions. We also find that learning is best predicted by a learner’s metacognitive appraisal of their knowledge gap, especially when the learner is on the verge of knowing. In contrast to the literature on curiosity, this effect is independent of curiosity, which raises the possibility that the cognitive effects of curiosity on learning might differ from those of metacognitive abilities. Whilst this study was based on an online sample of young adults, future research can extend this to other age groups to investigate the developmental changes underlying curiosity and metacognition (McGillivray et al., 2015). Thus, these results point to the critical importance of further investigations comparing the interplay of the mechanisms of curiosity and metacognition in human learning. Moreover, these results may also have implications for educational practice. For example, educators could explicitly provoke learners’ awareness of their knowledge gap to boost learning, whereas learners could frequently evaluate their knowledge levels to identify an intermediate gap to maximise learning (Twomey & Westermann, 2018; Kidd et al., 2012, 2014). Taken together, these findings provide new evidence for the role of curiosity and metacognition in learning, highlighting the role of metacognitive abilities in learning and a potential limitation of curiosity in learning.

## Electronic Supplementary Material

Below is the link to the electronic supplementary material.

Supplementary Material Table S1-S3

## Data Availability

No datasets were generated or analysed during the current study.
