# Supplementary material for: The limits of curiosity? New evidence for the roles of metacognitive abilities and curiosity in learning
Source: Metacogn Learn. 2024 Nov 15;20(1):1. doi: 10.1007/s11409-024-09407-9 (PMC12815978; doi:10.1007/s11409-024-09407-9)
Supplement: Supplementary file 1 — Supplementary Material Table S1-S3 [file 11409_2024_9407_MOESM1_ESM.docx]

**The Limits of Curiosity? New Evidence for the Roles of Metacognitive Abilities and Curiosity in Learning**

**Supplementary Information**

**Table S1.** *Variance Inflation Factors (VIF) for collinearity between variables in Model 1*

| **Variable** | **VIF** |
| --- | --- |
| Knowledge Confidence | 1.16 |
| Correctness Confidence | 1.35 |
| *Note:* VIF < 5 indicates no reason for concern about collinearity | |

**Table S2.** *VIF for collinearity between variables in the binomial generalised logistic mixed-effects model (Model 2)*

| **Variable** | **VIF** |
| --- | --- |
| Curiosity | 1.00 |
| Knowledge Confidence | 1.09 |
| Correctness Confidence | 1.19 |
| *Note:* VIF < 5 should not be a concern of collinearity | |

| **Table S3** | | | | |
| --- | --- | --- | --- | --- |
| *Estimates from the Comparison Model in predicting test recall accuracy* | | | | |
| Term | $\beta$ | *SE* | *z* | *p* |
| (Intercept) | 0.50 | 0.21 | 2.32 | .020* |
| CC | -0.04 | 0.10 | -0.43 | .667 |
| Curiosity | -0.04 | 0.12 | -0.31 | .754 |
| KC:Not Sure | 0.36 | 0.13 | 2.75 | .006** |
| KC:Yes | 0.20 | 0.17 | 1.15 | .249 |
| CC × Curiosity | -0.06 | 0.10 | -0.65 | .518 |
| CC × KC:Not Sure | 0.10 | 0.13 | 0.76 | .449 |
| CC × KC:Yes | 0.11 | 0.14 | 0.74 | .459 |
| Curiosity × KC:Not Sure | 0.14 | 0.13 | 1.02 | .306 |
| Curiosity × KC:Yes | 0.22 | 0.16 | 1.39 | .164 |
| CC × Curiosity × KC:Not Sure | 0.07 | 0.12 | 0.62 | .538 |
| CC × Curiosity × KC:Yes | -0.05 | 0.13 | -0.41 | .684 |
| *Note.* * p < .05, ** p < .01, *** p < .001; CC as Correctness Confidence, KC as Knowledge Confidence  *Comparison Model Structure:*  Recall Accuracy ~ Curiosity* Knowledge Confidence*Correctness Confidence + (1 + Correctness Confidence \| Participant) + (1 \| Stimulus), family = binomial  Summary: The model suggested that there were no significant interactions among curiosity and metacognitive appraisals in predicting recall accuracy (see Table S3 for summary). We also examined the significance of each predictor by dropping each predictor from this Comparison Model one at a time. None of the interaction terms were found as a significant predictor (*p* = .518). We also compared the Full model (presented in the initial manuscript) with the Comparison model, there was not significant improvement by including the interaction terms (*p* = 0.807). Overall, these results suggested that there were no significant interaction terms in predicting recall accuracy. Therefore, we reduced the Comparison Model to the Full Model 2 as in the initial manuscript. | | | | |
